# Two- and three-dimensional evaluation of endodontic microsurgery outcomes in maxillary anterior teeth with through-and-through lesions: a retrospective cohort study

**DOI:** 10.1186/s12903-025-07614-7

**Published:** 2026-01-03

**Authors:** Le Lu, Ke Xu, Ya Shen, He Liu

**Affiliations:** 1 Department of Endodontics, Suzhou Stomatological Hospital, No. 1366 Suzhan street, Suzhou, 215005 China; 2 Department of Oral Biological and Medical Sciences, Faculty of Dentistry, University of British Columbia, 2199 Wesbrook Mall, Vancouver, V6T 1Z3 Canada

**Keywords:** Endodontic microsurgery, Healing, Maxillary anterior teeth, Periapical lesion, Through-and-through

## Abstract

**Objective:**

This retrospective cohort study evaluated healing outcomes following endodontic microsurgery (EMS) in maxillary anterior teeth with through-and-through periapical lesions (PALs) using periapical radiographs and cone-beam computed tomography (CBCT).

**Methods:**

Permanent teeth with through-and-through PALs treated by EMS and followed for more than 24 months were included. Two calibrated endodontic specialists independently assessed two-dimensional (2D) healing according to the Molven criteria and three-dimensional (3D) healing using the PENN 3D criteria. Treatment outcomes were dichotomized as success (complete and incomplete/limited healing) or failure (uncertain and unsatisfactory healing). Preoperative and follow-up lesion volumes were calculated using Mimics software.

**Results:**

Sixteen patients (22 teeth, 16 through-and-through lesions) with a follow-up period ranging from 24 to 61 months (mean, 33 months) were included. Complete healing was observed in 62.5% of cases according to the 2D criteria and in 31.3% according to the 3D criteria. The mean lesion volume significantly decreased from 856.97 ± 566.06 mm³ preoperatively to 95.74 ± 180.45 mm³ at follow-up (*P* < 0.0001).

**Conclusions:**

Favorable healing outcomes were observed following EMS in maxillary anterior teeth with through-and-through lesions. CBCT-based assessment applied more stringent healing criteria than periapical radiographs and provided a more comprehensive evaluation of periapical bone regeneration.

**Supplementary Information:**

The online version contains supplementary material available at 10.1186/s12903-025-07614-7.

## Introduction

Endodontic microsurgery (EMS) is a contemporary surgical approach that integrates magnification, illumination, and microsurgical instruments for the management of periapical pathoses while preserving surrounding tissues [1–3]. Compared with conventional periapical surgery, EMS allows more precise root-end management, less postoperative discomfort, and improved healing outcomes [1–3]. In addition, the use of guided tissue regeneration (GTR) techniques and adjunctive biomaterials, such as collagen membranes, bone grafts, and platelet-derived products, has been introduced to enhance periapical bone regeneration in selected cases [4–6].

Through-and-through PALs in maxillary anterior teeth remain particular challenging. These defects involve perforation of both the buccal and palatal cortical plates and are often associated with extensive bone loss and an increased risk of delayed healing or postoperative complications [7, 8]. The absence of a bony barrier may facilitate soft-tissue ingrowth and compromise bone regeneration. In the esthetic zone, treatment failure may result in esthetic concerns and complicate future implant placement or restorative rehabilitation.

Healing outcomes following EMS has traditionally been evaluated using two-dimensional (2D) periapical radiographs based on Molven criteria [9]. However, 2D imaging has inherent limitations in assessing buccolingual bone changes and cortical plates integrity. Cone-beam computed tomography (CBCT) enables three-dimensional (3D) assessment of periapical healing using the PENN 3D criteria [10, 11], providing more detailed visualization of bone regeneration at the resection plane, apical region, and surrounding cortical plates.

Although several studies have reported clinical outcomes of EMS in through-and-through lesions, data focusing specifically on maxillary anterior teeth remain limited, and dynamic volumetric changes assessed by CBCT have not been well characterized [12–15]. Therefore, the aim of the present study was to evaluate healing outcomes following EMS in maxillary anterior teeth with through-and-through PALs using both 2D and 3D radiographic criteria, and to quantify preoperative and postoperative lesion volumes to assess periapical bone regeneration.

## Materials and methods

### Study design and ethics

**Fig. 1:**
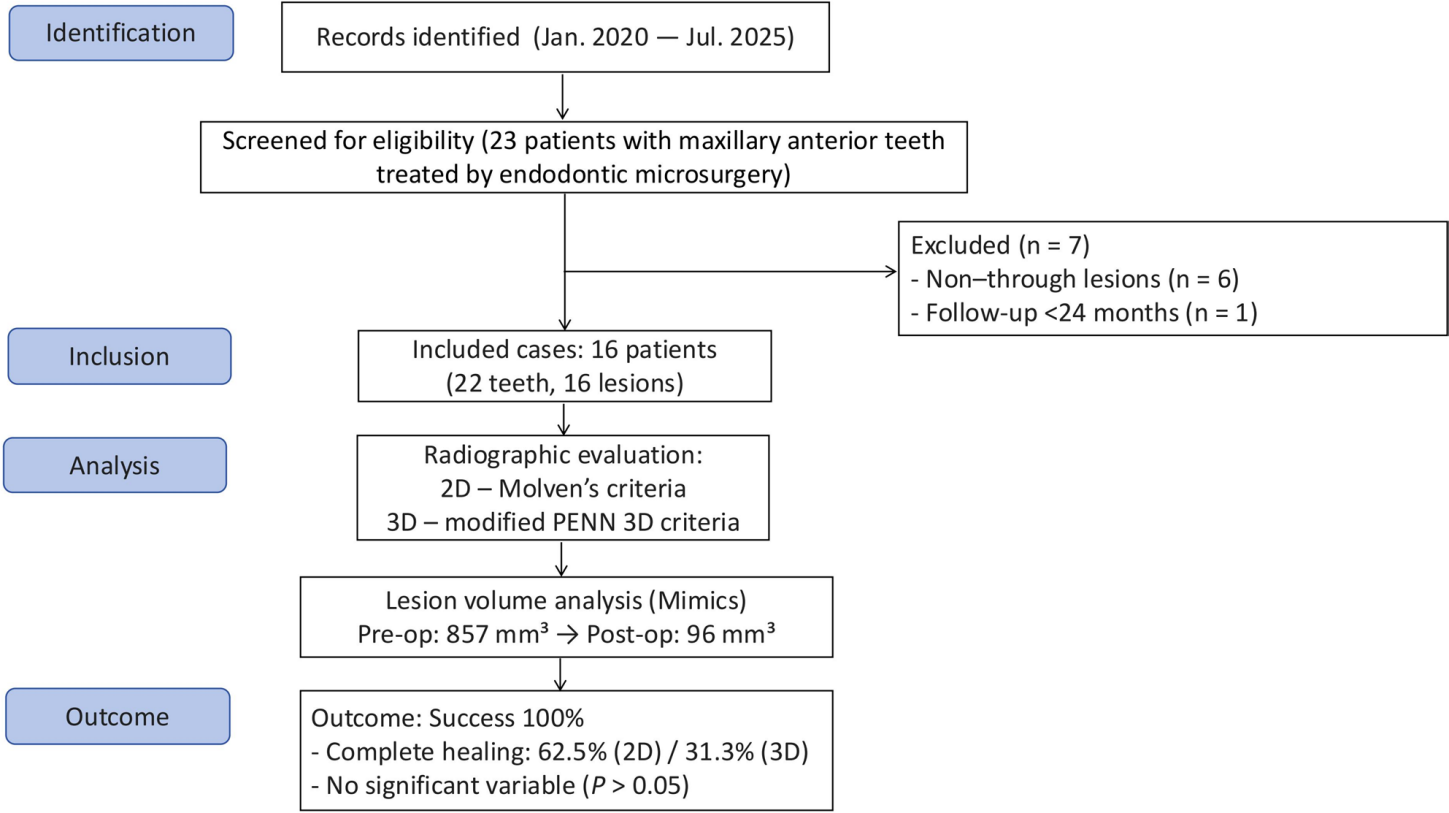
A flow diagram summarizing the processes of participant screening, eligibility evaluation, exclusion, and final inclusion

This retrospective cohort study was approved by the Research Ethics Committee of Suzhou Stomatological Hospital (approval No. SZKQYY-2020-H003). This study was conducted in compliance with the Strengthening the Reporting of Observational Studies in Epidemiology (STROBE) guidelines [16]. A flow diagram summarizing the processes of participant screening, eligibility evaluation, exclusion, and final inclusion is presented in Fig. 1.

### Participants

The participants in this study were patients with maxillary anterior teeth presenting with through-and-through PALs who were treated with EMS. Dental records were retrieved from the hospital database between January 2020 and July 2025. The inclusion criteria were as follows: (1) maxillary anterior teeth diagnosed with through-and-through PALs (defined as lesions perforating both the buccal and palatal cortical plates); (2) a minimum follow-up period of 24 months; and (3) complete preoperative, intraoperative, and postoperative clinical and radiographic records, including PAs and CBCT scans. The exclusion criteria were as follows: non–through-and-through lesions; teeth diagnosed preoperatively or intraoperatively with vertical root fracture (VRF); a follow-up period of less than 24 months; or incomplete clinical or radiographic documentation. All participants provided written informed consent at the time of treatment, agreeing to the use of their anonymized clinical data for research purposes.

### Clinical and radiographic examination and diagnosis

All teeth underwent comprehensive clinical and radiographic examination. Clinical assessment included periodontal probing, percussion and palpation tests, evaluation of tooth mobility, and inspection for sinus tracts. At preoperative examination, no included teeth exhibited isolated periodontal probing depths greater than 4 mm. Preoperative periapical radiographs and CBCT scans were obtained to assess root morphology and length, the location and extent of PALs, and their relationship with adjacent anatomical structures. Teeth with suspected VRF or root cracks were excluded based on a standardized clinical and radiographic evaluation protocol. Clinically, teeth presenting with features suggestive of VRF—such as isolated deep periodontal probing depths, persistent sinus tracts, or characteristic J-shaped bone loss—were excluded. Radiographically, CBCT images were evaluated under standardized viewing conditions for fracture lines, halo-like or longitudinal radiolucencies extending along the root surface, or bone loss patterns inconsistent with lesions of endodontic origin. Only teeth without clinical or CBCT findings indicative of VRF were included in the study.

### Surgical procedure

All surgical procedures were performed by the same experienced endodontist under magnification and illumination using a dental operating microscope (Zumax 2380, Suzhou, China). Preoperative intraoral and extraoral disinfection was carried out using standard antiseptic protocols, followed by local anesthesia with 4% articaine containing 1:100,000 epinephrine.

A full-thickness mucoperiosteal flap (labial/buccal or palatal) was designed based on the location and extent of the osseous defect. The incision extended through the attached gingiva, mucosa, and periosteum to expose the underlying bone. After flap elevation, the submucosa was carefully separated from the periosteum to to provide adequate surgical access while minimizing soft tissue trauma.

Osteotomy was performed under continuous sterile saline irrigation using an ImpactAir45 contra-angle handpiece (Palisades Dental, Englewood, NJ, USA) with a high-speed fissure bur. Granulation tissue was thoroughly curetted and submitted for histopathological examination. Apicoectomy was carried out by resecting approximately 3 mm of the root apex perpendicular to the long axis of the root to eliminate apical ramifications and lateral canals. Hemostasis was achieved as needed using sterile cotton pellets soaked in epinephrine solution (1:1,000), applied locally for short durations to improve visualization of the surgical field.

The resected root surface was stained with 1% methylene blue solution and carefully inspected under high magnification to detect anatomical details and to exclude the presence of vertical root fractures (VRF) or root cracks. A 3-mm-deep root-end cavity was prepared using ultrasonic tips under copious irrigation. The root-end cavity was gently dried using sterile paper points and subsequently filled with iRoot BP Plus (Innovative Bioceramix, Vancouver, Canada), achieving a dense, well-adapted, and void-free root-end seal.

The GTR technique was performed in eight cases. In five cases, both buccal and palatal flaps were reflected, and the osseous defect was covered with a resorbable collagen membrane (Hyundai Bioland Co., Ltd., Cheongju, Korea). In the remaining three cases, only the buccal flap was reflected and covered with the membrane. Following completion of all surgical procedures, the flap was repositioned carefully and sutured. Postoperative periapical radiographs were taken to confirm the quality and extent of the root-end filling.

Postoperatively, patients were prescribed antibiotics and analgesics as needed and instructed to rinse with 0.2% chlorhexidine gluconate mouthwash for three days. Sutures were removed one week after surgery. During follow-up visits, patients were interviewed regarding postoperative symptoms, underwent clinical examinations, and received periapical radiographs and CBCT imaging to assess periapical healing.

### Outcome assessment

**Fig. 2:**
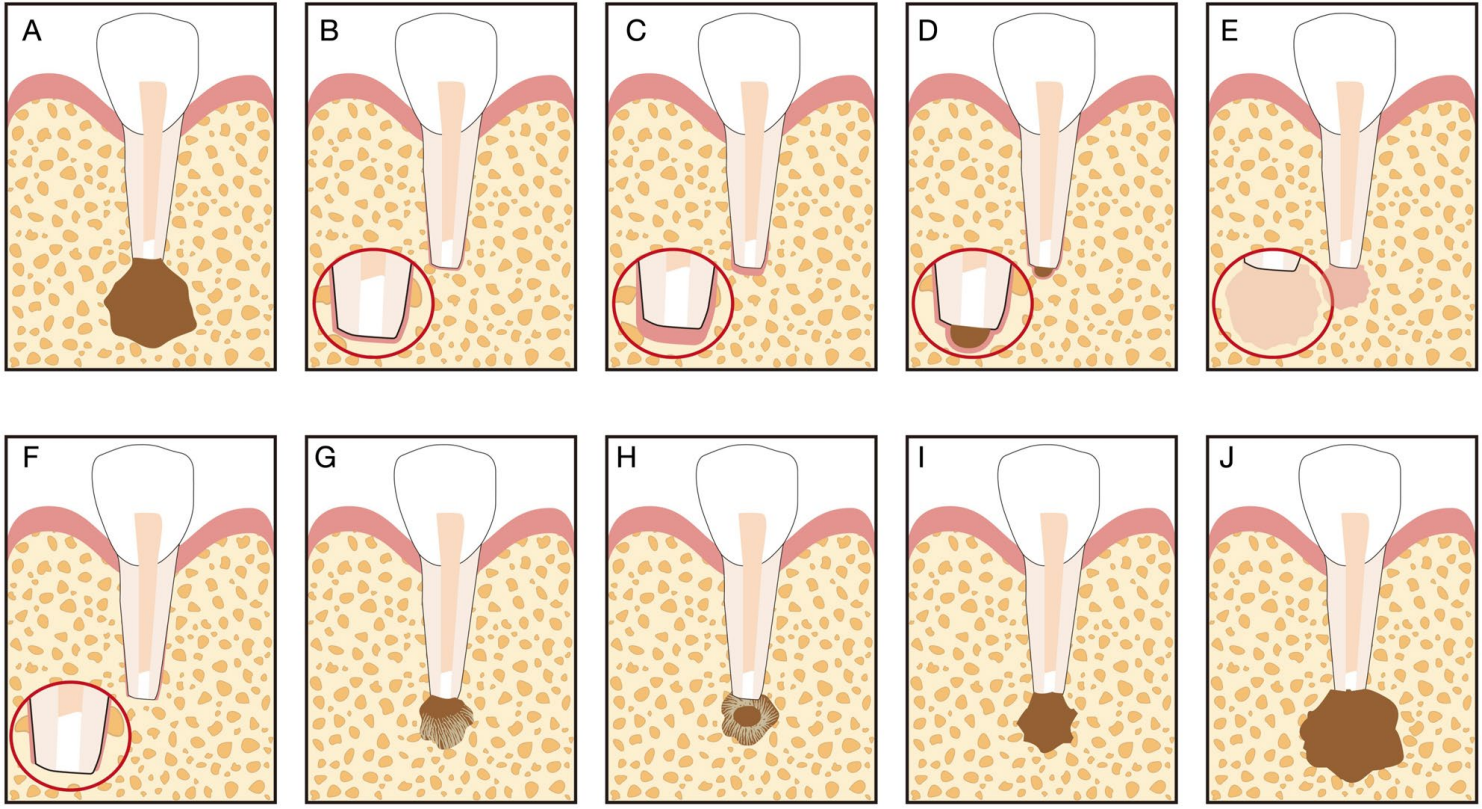
Schematic illustration of the 2D Molven criteria. **A** Immediate postoperative radiograph. **B**–**F** Complete healing. **G**, **H** Incomplete or limited healing. **I** Uncertain healing. **J** Unsatisfactory healing. Adapted with permission from Ref [15]

**Fig. 3:**
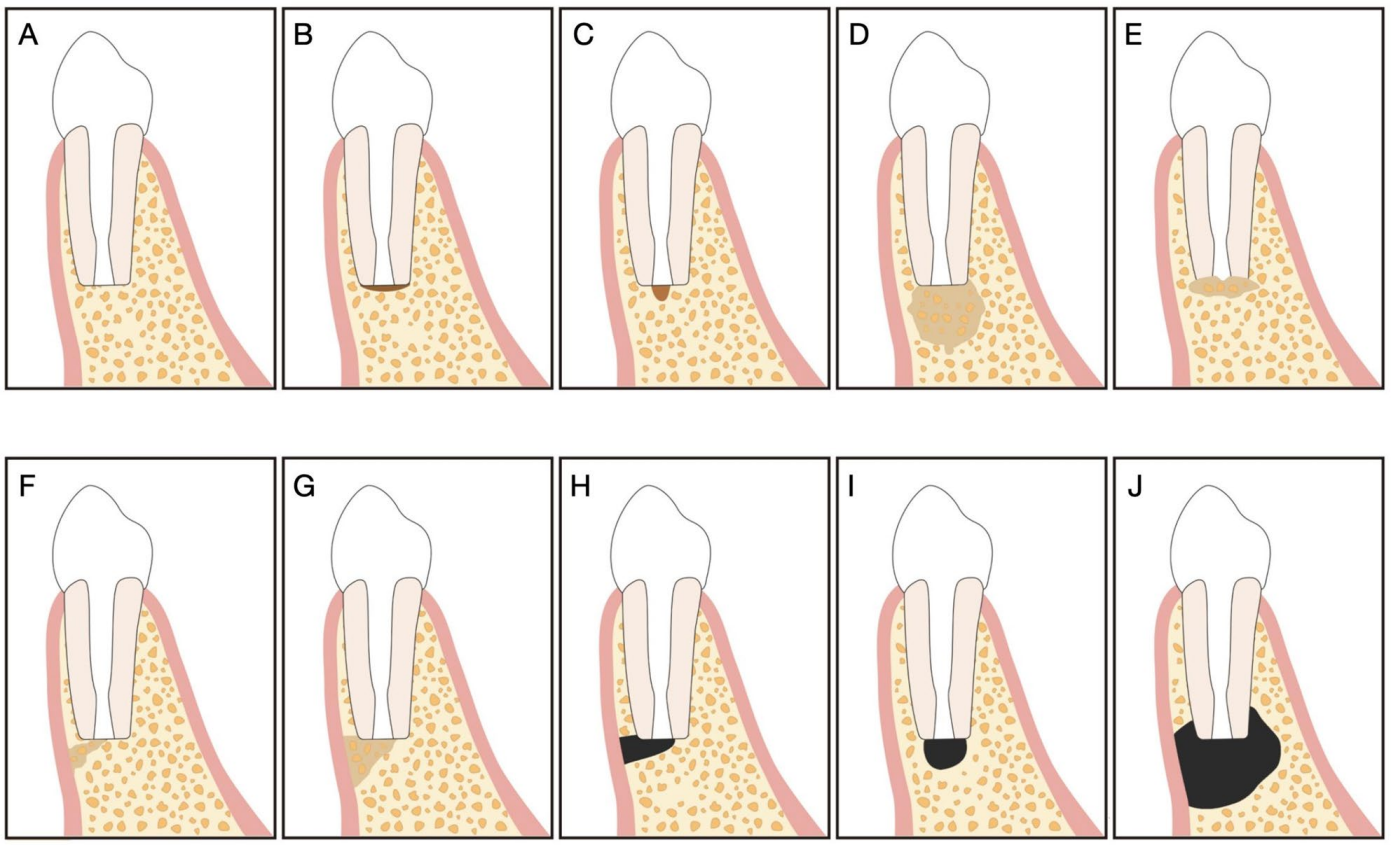
Schematic illustration of the 3D PENN criteria. **A**–**E** Complete healing. **F**-**I** limited healing. **J** Unsatisfactory healing. Adapted with permission from Ref [17]

Two calibrated examiners independently evaluated all radiographic images. Healing on periapical radiographs was assessed according to 2D Molven criteria (Fig. 2) [9, 15], whereas 3D healing on CBCT scans was evaluated using the 3D PENN criteria (Fig. 3) [10, 11, 17] (Table 1).

**Table 1:** Criteria for periapical radiographs and CBCT assessment of healing

Category	2D Molven Criteria (Periapical Radiographs)	3D Penn Criteria (CBCT)
Complete healing	• Periodontal ligament (PDL) space restored to normal width or lamina dura reconstituted around the apex (≤ 2× normal width). • Tiny lamina dura defect (< 1 mm²) adjacent to filling tolerated. • Bony cavity filled with bone, though radiopacity may differ from surrounding bone.	• A normal-width PDL space and an intact lamina dura are restored across both resected and unresected root surfaces. • The apical PDL over the resected surface may be mildly wider, but never more than twice the width seen on unaffected root areas. • A small break in the lamina dura can be present around the root-end filling. • Bone fill is complete with a visible lamina dura; bone next to the apex can remain slightly less dense than surrounding uninvolved bone. • Hard tissue completely covers the resected root end, and no apical PDL space is detectable.
Incomplete / Limited healing	• Radiolucent area decreased in size or stationary with bone healing at periphery. • Bone structure may appear within rarefaction; typically asymmetric or angular around apex.	• Healing is evident, yet cortical continuity is interrupted by a focal low-density area. • A residual low-density zone persists asymmetrically around the apex or joins the PDL space at an angle. • Considerable bone has formed, but the prior access/osteotomy site has not fully ossified. • The cortical plate appears re-established, but a low-density region remains adjacent to the resected root surface.
Uncertain healing	• Reduced radiolucency but > 2× normal PDL width. • May or may not be bordered by lamina dura–like bone. • Circular/semi-circular periphery symmetrically around apex (funnel-shaped PDL	
Unsatisfactory healing	• Radiolucency unchanged or increased in size.	• The low-density area is unchanged or larger in volume, indicating absent or poor healing.

Prior to the formal evaluation, both examiners were calibrated using 10 periapical radiographs and 10 CBCT scans not included in the study. The calibration process involved independent assessments followed by consensus discussions based on the predefined 2D and 3D criteria. To evaluate reliability, the same set of images was re-examined by each examiner after a two-week interval. Intra-examiner and inter-examiner reliability were analyzed using Cohen’s kappa statistics (see Sect. 2.67).

Any discrepancies identified during the final evaluation were resolved through discussion until consensus was achieved. Treatment outcomes were classified as either success or failure. Success was defined as the absence of clinical symptoms and the presence of radiographic evidence of complete (2D/3D), incomplete (2D), or limited (3D) healing. Failure was defined as the presence of clinical symptoms and/or radiographic findings consistent with uncertain or unsatisfactory healing.

### Calculation of preoperative and follow-up lesion volumes

Preoperative and follow-up volumes of the PALs were calculated using Mimics Medical software version 21.0 (Materialise, Leuven, Belgium). CBCT DICOM datasets were anonymized and imported with identical voxel size and field-of-view settings for baseline and follow-up scans [18]. A region of interest (ROI) encompassing the lesion and surrounding cortical plates was defined for each case.

**Fig. 4:**
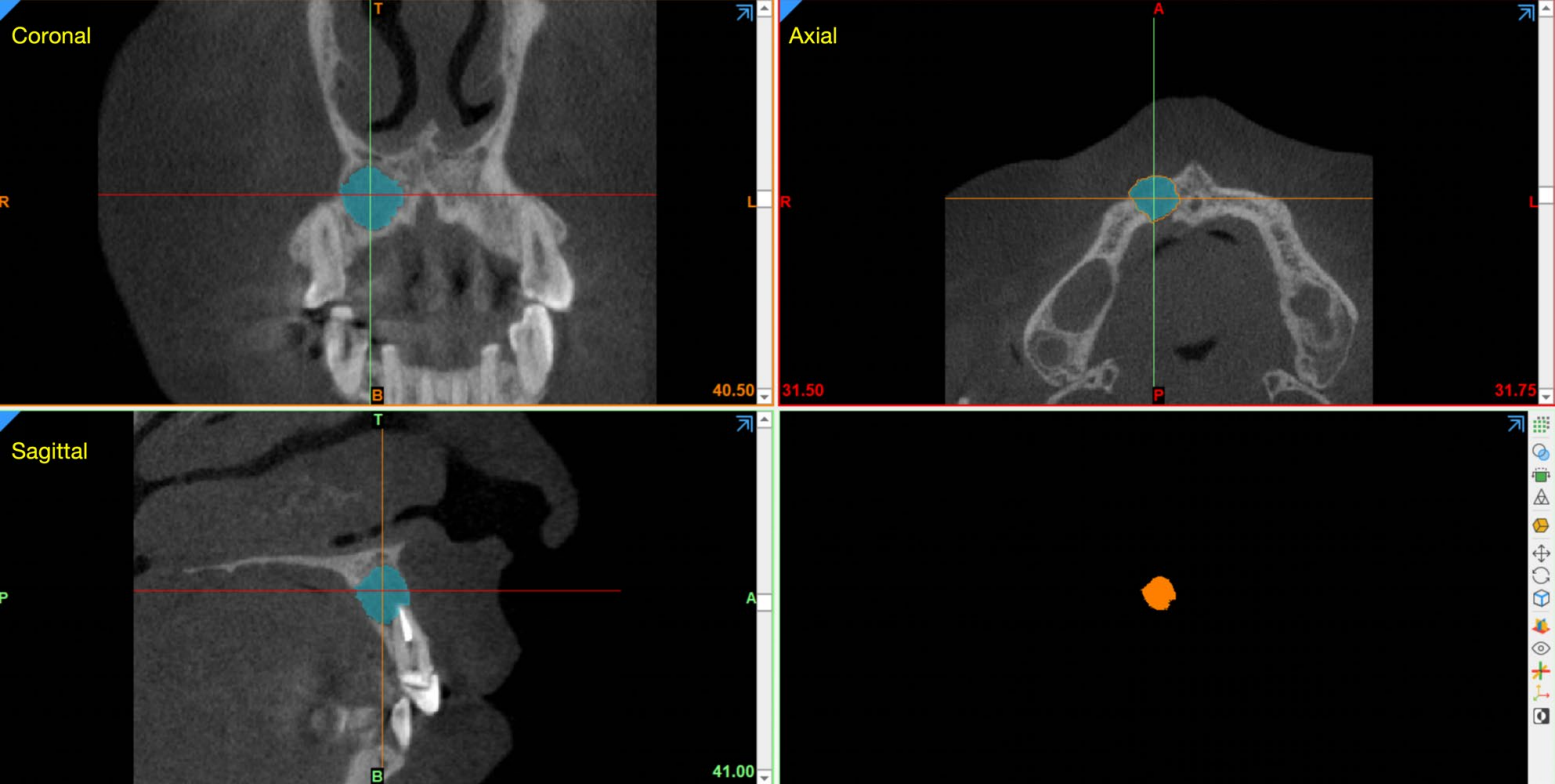
Rendering, 3D reconstruction, and calculation of lesion volume for a through-and-through lesion using Mimics software

Lesion segmentation was performed using a semi-automated, threshold-based region-growing approach with grayscale threshold levels determined based on visual inspection of lesion boundaries and surrounding bone. Consistent window and level presets were applied across all cases to ensure comparability. Following automatic segmentation, slice-by-slice manual refinement was conducted in the axial, coronal, and sagittal planes to exclude root canals, periodontal ligament space, and postoperative voids. Three-dimensional reconstructions were then generated, and lesion volume (mm³) was calculated using the “Calculate 3D Properties” tool (Fig. 4). The same segmentation protocol and parameters were applied to both preoperative and follow-up scans.

To minimize segmentation variability, all volumetric measurements were performed by a single calibrated examiner with experience in CBCT analysis. A subset of randomly selected cases was re-segmented after a two-week interval, and the measurements were compared to assess intra-observer reliability.

### Statistical analysis

Statistical analysis was performed using GraphPad Prism version 10.0.2 (GraphPad Software Inc., San Diego, CA, USA). Continuous variables are presented as mean ± standard deviation (SD), and categorical variables as frequency and percentage. Categorical variables were analyzed using Fisher’s exact test, while preoperative and postoperative lesion volumes were compared using a paired t-test. Inter- and intra-examiner agreement for radiographic assessments were evaluated using Cohen’s kappa (κ) statistics, with values interpreted as follows: <0.40, poor agreement; 0.41–0.60, moderate; 0.61–0.80, good; and > 0.80, excellent agreement. A *P* value < 0.05 was considered statistically significant.

## Results

A total of 23 patients were initially screened for eligibility. Seven patients were excluded—six due to non–through-and-through lesions and one due to a follow-up period of less than 24 months. Ultimately, 16 patients with 16 through-and-through PALs involving 22 teeth and a follow-up period ranging from 24 to 61 months (mean, 33 months) were included in the final analysis. All included patients were classified as American Society of Anesthesiologists (ASA) physical status I, had no systemic diseases, and reported no relevant medication use.

Both examiners independently re-evaluated 10 periapical radiographs and 10 CBCT scans after a two-week interval. Intra-examiner agreement was 100% for both examiners (κ = 1.00, *P* < 0.001), indicating perfect reproducibility. Inter-examiner agreement was 90% in both evaluation rounds (κ = 0.88, *P* < 0.001), demonstrating excellent consistency between examiners.

**Fig. 5:**
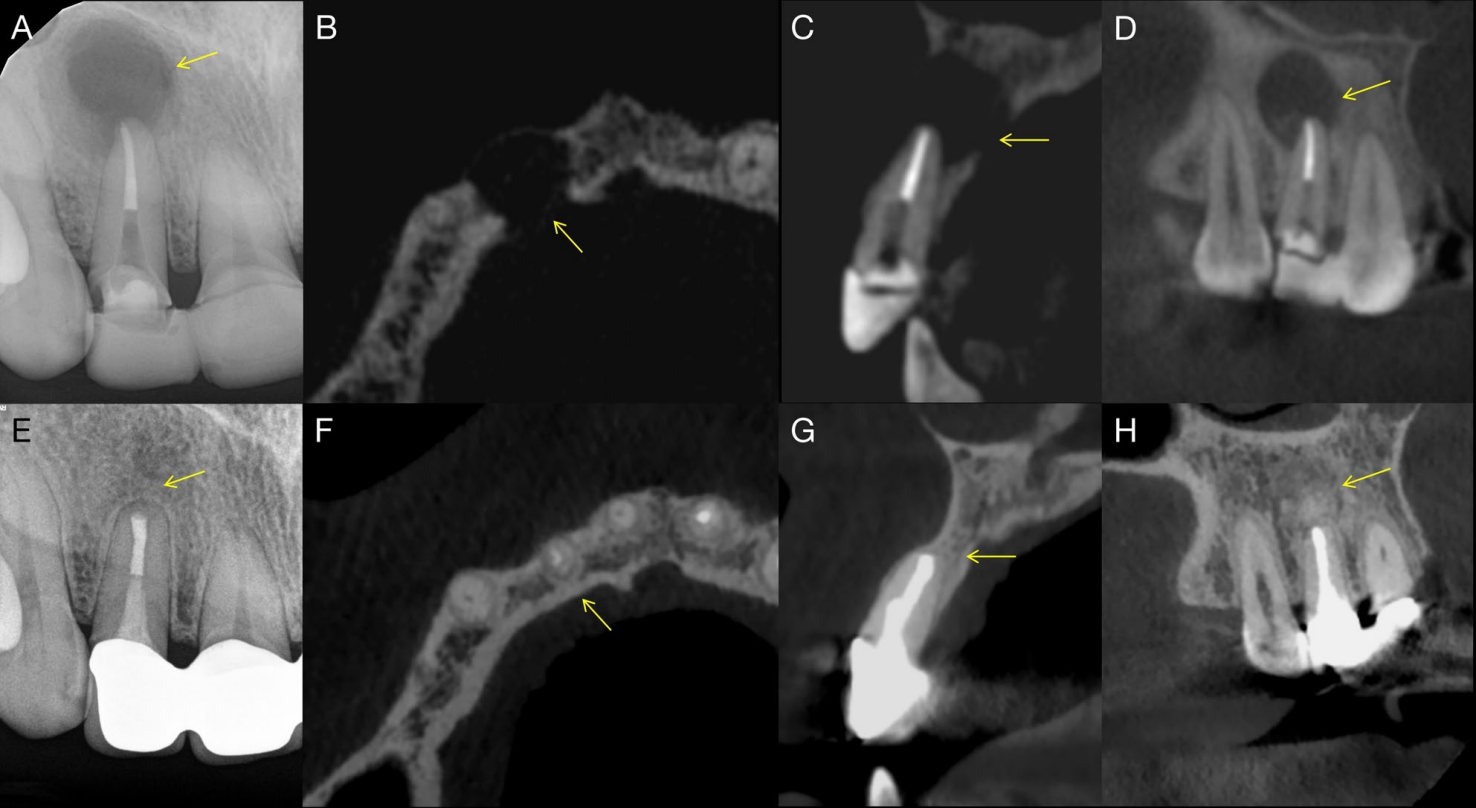
Complete healing of a through-and-through periapical lesions after endodontic microsurgery. **A** Preoperative periapical radiograph showing a large radiolucent lesion associated with the maxillary right central incisor (arrow). **B** Axial CBCT slice demonstrating a through-and-through bone defect perforating both cortical plates (arrow). **C** Sagittal CBCT slice showing extensive periapical bone loss around the root apex (arrow). **D** Coronal CBCT slice confirming destruction of both buccal and palatal cortices (arrow). **E** Follow-up periapical radiograph showing complete bone fill at the surgical site (arrow). **F** Axial CBCT slice demonstrating full cortical plate regeneration (arrow). **G** Sagittal CBCT slice revealing continuous trabecular bone formation at the resection plane (arrow). **H** Coronal CBCT slice showing re-established cortical integrity and complete bone healing (arrow)

**Fig. 6:**
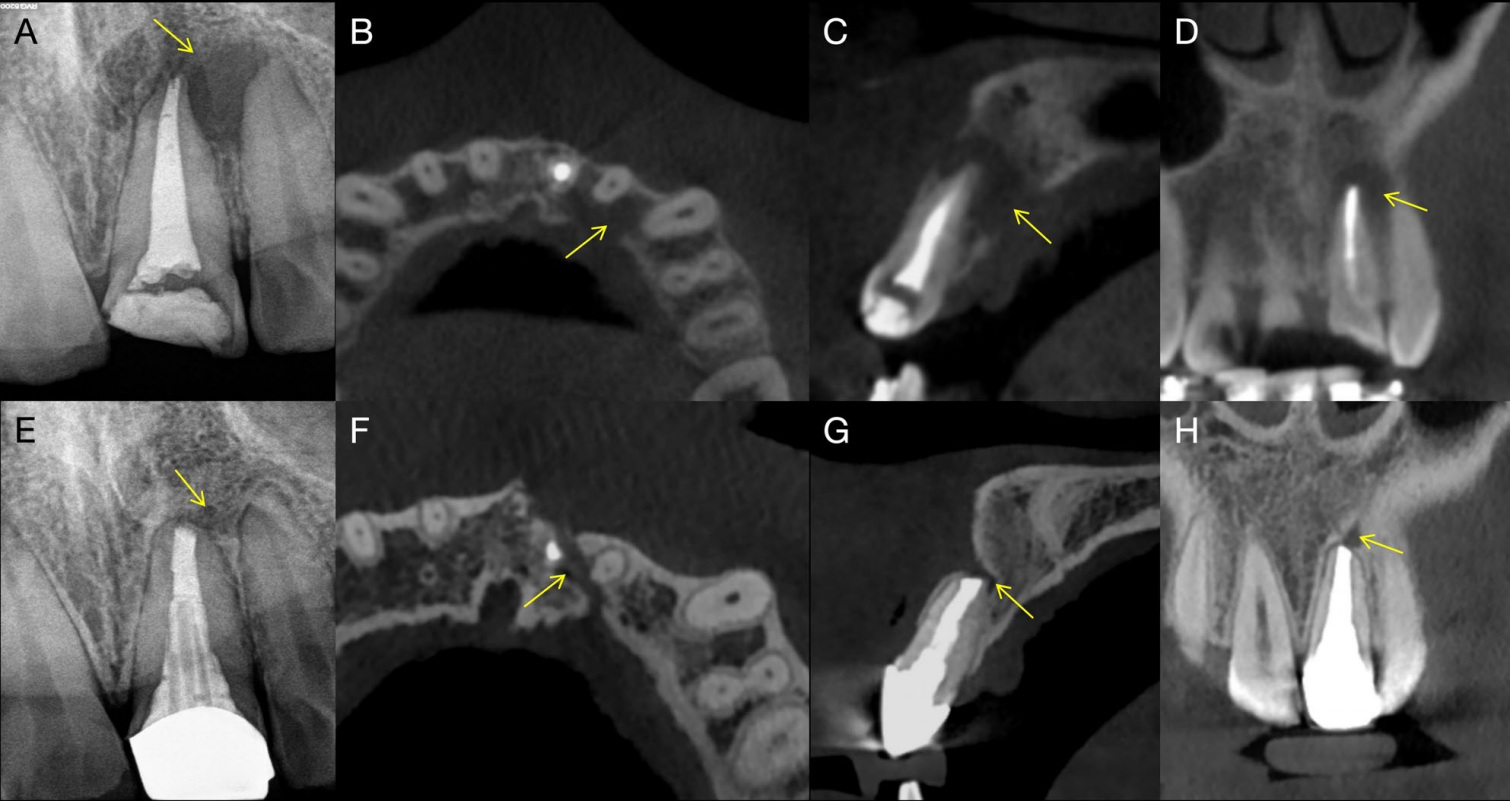
Incomplete healing of a through-and-through periapical lesions after endodontic microsurgery. **A** Preoperative periapical radiograph revealing a radiolucent lesion associated with the maxillary left central incisor involving both buccal and palatal cortical plates (arrow). **B** Axial CBCT slice demonstrating cortical plate perforation on both sides (arrow). **C** Sagittal CBCT slice showing extensive bone loss surrounding the root apex (arrow). **D** Coronal CBCT slice confirming complete perforation through buccal and palatal cortices (arrow). **E** Follow-up periapical radiograph showing partial bone fill at the surgical site (arrow). **F** Axial CBCT slice revealing bone regeneration with residual cortical discontinuity (arrow). **G** Sagittal CBCT slice indicating incomplete bone regeneration near the resection plane (arrow). **H** Coronal CBCT slice showing persistent cortical defect on the palatal aspect (arrow)

Healing outcomes were assessed using the 2D Molven and 3D PENN criteria. Table 2 summarizes the evaluated variables, including demographic characteristics, smoking status, patient symptoms, and treatment-related factors, together with their associated healing outcomes. According to the 2D Molven criteria, 10 cases (62.5%) were classified as complete healing and 6 cases (37.5%) as incomplete or limited healing. Based on the 3D PENN criteria, 5 cases (31.3%) demonstrated complete healing, while 11 cases (68.7%) showed limited healing. No cases were classified as uncertain or unsatisfactory healing according to either the 2D Molven or the 3D PENN criteria; notably, all cases classified as incomplete or limited healing were clinically asymptomatic at follow-up, with no pain, swelling, sinus tract formation, or tenderness to percussion or palpation. No statistically significant associations were identified between the evaluated variables and healing outcomes (*P* > 0.05). Representative cases of complete (Fig. 5) and incomplete (Fig. 6) healing following EMS are presented.

**Table 2:** Preoperative and intraoperative variables and healing outcomes

Variables	*n* (%)		2-D Molven criteria				*P* value		3D Penn criteria			*P* value
			Complete healing		Incomplete/limited healing				Complete healing		Limited healing	
Sex												
Male	8 (50%)		4		4		0.6087		1		7	0.2816
Female	8 (50%)		6		2				4		4	
Age												
≤ 25 years	8 (50%)		6		2		0.6084		4		4	0.2821
> 25 years	8 (50%)		4		4				1		7	
Smoking												
Yes	3 (18.75%)		1		2		0.5179		0		3	0.5089
No	13 (81.25%)		9		4				5		8	
Swelling												
Present	7 (43.75%)		5		2		0.6329		3		4	0.5962
Absent	9 (56.25%)		5		4				2		7	
Sinus tract												
Present	8 (50%)		5		3		> 0.9999		1		7	0.2821
Absent	8 (50%)		5		3				4		4	
Lesion volume (mm^3^)												
≤ 800	9 (56.25%)		7		2		0.3024		3		6	> 0.9999
> 800	7 (43.75%)		3		4				2		5	
GTR												
Yes	8 (50%)		5		3		> 0.9999		2		6	> 0.9999
No	8 (50%)		5		3				3		5	

Table 3 presents the preoperative and follow-up lesion volumes of through-and-through PALs. The mean preoperative lesion volume was 856.97 ± 566.06 mm³ (range, 250.82–2563.12 mm³), which significantly decreased to 95.74 ± 180.45 mm³ (range, 0–618.42 mm³) at follow-up (*P* < 0.0001).

**Table 3:** Preoperative and follow-up lesion volumes of through-and-through lesions

Time point	Lesion volume, mm³ (mean ± SD, range)	*P* value
Preoperative	856.97 ± 566.06 mm³ (250.82–2563.12)	< 0.0001
Follow-up	95.74 ± 180.45 mm³ (0–618.42)	

## Discussion

Management of through-and-through PALs in maxillary anterior teeth remains a significant clinical challenge. These lesions are frequently associated with extensive cortical bone loss on both the buccal and palatal sides, which can complicate surgical access, debridement, and postoperative healing. Compared with localized PALs, such defects are generally associated with a less favorable prognosis. Treatment failure in this region may result in esthetic compromise, alveolar bone deficiency, and increased difficulty in subsequent implant placement or restorative rehabilitation. In recent years, EMS with or without GTR techniques and adjunctive biomaterials, has been increasingly used to manage extensive periapical defects. The present study aimed to evaluate healing outcomes following EMS in maxillary anterior teeth with through-and-through PALs using both 2D and 3D radiographic criteria, and to explore factors potentially associated with healing.

In the present study, healing outcomes following EMS were assessed by both 2D Molven and 3D PENN criteria. Although healing was observed across cases, the proportion of complete healing differed substantially between assessment methods, with 62.5% classified complete healing according to the 2D criteria and 31.3% according to the 3D criteria. This discrepancy highlights the greater stringency of CBCT-based evaluation. PRs remain a convenient tool for routine clinical follow-up; however, their limitations in accessing buccolingual bone regeneration and cortical plate integrity are well recognized. In contrast, CBCT enables volumetric visualization of the resection plane, apical region, and both cortical plates, allowing a more comprehensive evaluation of bone healing. These findings are consistent with previous reports indicating that 3D assessment is more sensitive in detecting residual bone defects and may better reflect the biological completeness of healing following EMS [19].

Healing was observed in the included cases despite the challenging nature of through-and-through lesions, which are often associated with extensive bone loss and a guarded prognosis. Azim et al. reported a success rate of 78.6% in a 3D retrospective analysis of 14 through-and-through lesions [12]. Yang et al. reported a success rate of 94.1% and a complete healing rate of 82.4% in a 2D retrospective study of 77 cases [15]; however, the number of maxillary anterior teeth included in that cohort was not specified. The favorable healing outcomes observed in the present study should be interpreted with caution, as they may have been influenced by careful case selection, treatment performed by an experienced operator, and the retrospective study design. Nevertheless, the results are in line with existing evidence suggesting that contemporary microsurgical techniques, such as the used of imagnification, ultrasonic root-end preparation, and bioceramic root-end filling materials, may contribute to improved healing in through-and-through lesions, even in the presence of large defects or challenging anatomical conditions.

The success of EMS is influenced by multiple factors, including patient age, tooth type, lesion size, preoperative symptoms, surgical technique, and the use of regenerative adjuncts [2–4]. In the present study, no statistically significant associations were observed between the evaluated demographic or clinical variables—such as sex, age, preoperative swelling, sinus tract, lesion volume, or use of GTR—and healing outcomes. However, these findings should be interpreted with caution, as the limited sample size reduced the statistical power to detect potential effects. The standardized surgical protocol and treatment by an experienced operator may also have reduced variability among cases. These observations are consistent with the findings of Parmar et al., who reported that EMS, with or without GTR, resulted in favorable outcomes for through-and-through lesions, with no additional benefit associated with collagen membrane use [5]. Other studies have reported mixed results regarding regenerative adjuncts, with platelet-rich plasma showing potential benefits in some through-and-through lesions [6], whereas injectable platelet-rich fibrin combined with type I collagen particles did not demonstrate improved bone regeneration in similar defects [14].

GTR may serve as a useful adjunct to EMS in cases presenting with marginal periodontal attachment loss; however, differentiation between periodontal breakdown of endodontic origin and that associated with VRF is essential [20–22]. Periodontal defects secondary to endodontic lesions are typically localized and apically oriented, whereas VRF-related lesions more often present as deep, narrow, and J-shaped or halo-like radiolucencies extending along the root surface and are frequently associated with isolated deep periodontal probing depths [22]. The extent and configuration of bone defects observed on CBCT imaging can therefore aid in identifying the primary etiology of endodontic failure following primary root canal treatment [23]. In the present study, careful clinical and CBCT-based evaluation was used to exclude cases with suspected VRF, supporting appropriate case selection for EMS with or without GTR.

CBCT plays an important role in the preoperative, intraoperative, and postoperative evaluation of EMS [23, 24]. The 3D imaging facilitates accurate diagnosis, surgical planning, and assessment of healing, particularly in complex cases such as large through-and-through lesions or lesions in proximity to vital anatomical structures. Image acquisition should adhere to the ALARA principle (As Low As Reasonably Achievable), using a limited field of view and optimized exposure parameters. Quantitative CBCT analysis in the present study demonstrated a significant reduction in lesion volume following surgery, indicating substantial periapical bone regeneration. Volumetric analysis provides an objective adjunct to categorical healing assessments and may assist in identifying cases with delayed or incomplete healing that warrant closer monitoring or intervention [18]. It should be noted that 2D radiographic outcome assessment can achieve high reproducibility when calibrated systems such as Ørstavik’s periapical index (PAI), based on a reference set of 100 radiographs, are used [25]. Nevertheless, CBCT-based evaluation provides improved diagnostic accuracy and 3D characterization of periapical healing, particularly in complex lesions, and therefore served as a complementary and more comprehensive assessment method in the present study.

From a clinical perspective, although both periapical radiographs and CBCT allow longitudinal evaluation of periapical healing, true dynamic or late healing leading to complete reossification appears uncommon beyond a minimum follow-up period of two years after EMS. Because inflammatory and granulation tissues are thoroughly removed during surgery, radiographic findings classified as incomplete or limited healing at long-term follow-up are more likely to represent stable postoperative conditions rather than ongoing reparative processes. Neither imaging modality reliably indicates subsequent progression to complete reossification in such cases. In the present study, lesions categorized as incomplete or limited healing remained clinically asymptomatic and showed no evidence of progressive pathology, supporting their interpretation as stable outcomes.

This study has several limitations. First, the exclusive inclusion of maxillary anterior teeth may limit the generalizability of the findings to other tooth types, although this approach reduced potential confounding related to tooth position. Second, although dynamic CBCT-based volumetric analysis of through-and-through periapical lesions allowed quantitative assessment of bone regeneration over time and provided greater objectivity than conventional two-dimensional evaluations, this imaging-based approach remains subject to inherent methodological constraints. In addition, the sample size was relatively small, and the retrospective design limited control over potential confounding factors, such as lesion size, surgical technique, and operator-related variability. The small sample size also reduced the statistical power; therefore, findings related to prognostic factors should be interpreted with caution. Furthermore, the analysis was performed at the lesion level rather than the individual tooth level, which may limit the applicability of the results to posterior teeth or cases with more complex root morphology. Finally, the use of postoperative antibiotics and a standardized follow-up protocol may have influenced healing patterns and may limit generalizability to clinical settings employing different postoperative management strategies.

Future studies with larger sample sizes, prospective designs, and multicenter participation are needed to validate these findings and further explore prognostic factors. Randomized clinical trials comparing outcomes between through-and-through and non–through-and-through lesions, as well as studies evaluating the role of regenerative adjuncts, may help refine treatment strategies for extensive periapical defects.

## Conclusion

EMS was associated with favorable healing outcomes in maxillary anterior teeth with through-and-through lesions. CBCT-based assessment applied more stringent healing criteria than periapical radiographs and provided a more comprehensive evaluation of periapical bone regeneration. The 3D imaging and volumetric analysis may therefore serve as valuable adjuncts for the assessment of healing in complex PALs. Acknowledgements.

## Supplementary Information

Supplementary Material 1.

## Data Availability

The datasets used and/or analyzed during the current study are available from the corresponding author, Dr. He Liu (email: he.liu@ubc.ca), upon reasonable request.

## Abbreviations

EMS: Endodontic microsurgery
CBCT: Cone-beam computed tomography
2D: Two-dimensional
3D: Three-dimensional
PDL: Periodontal ligament
PAL: Periapical lesion
GTR: Guided tissue regeneration
